# The Effects of High-Protein Diet and Resistance Training on Glucose Control and Inflammatory Profile of Visceral Adipose Tissue in Rats

**DOI:** 10.3390/nu13061969

**Published:** 2021-06-08

**Authors:** Claudia Stela Medeiros, Ivo Vieira de Sousa Neto, Keemilyn Karla Santos Silva, Ana Paula Castro Cantuária, Taia Maria Berto Rezende, Octávio Luiz Franco, Rita de Cassia Marqueti, Leandro Ceotto Freitas-Lima, Ronaldo Carvalho Araujo, Azize Yildirim, Richard Mackenzie, Jeeser Alves Almeida

**Affiliations:** 1 Programa de Pós-Graduação em Saúde e Desenvolvimento na Região Centro-Oeste, Faculdade de Medicina, Universidade Federal de Mato Grosso do Sul, Campo Grande 79070-900, Brazil; claudiastela2@gmail.com; 2 Laboratório de Análises Moleculares, Programa de Pós-Graduação em Ciências e Tecnologias em Saúde, Universidade de Brasília, Distrito Federal 72220-275, Brazil; ivoneto04@hotmail.com (I.V.d.S.N.); marqueti@gmail.com (R.d.C.M.); 3 Research in Exercise and Nutrition in Health and Sports Performance—PENSARE, Graduate Program in Movement Sciences, Universidade Federal de Mato Grosso do Sul, Campo Grande 79070-900, Brazil; keemilyn.karla@gmail.com; 4 Centro de Análises Proteômicas e Bioquímicas, Programa de Pós-Graduação em Ciências Genômicas e Biotecnologia, Universidade Católica de Brasília, Distrito Federal 70790-160, Brazil; apccantuaria@gmail.com (A.P.C.C.); taiambr@gmail.com (T.M.B.R.); ocfranco@gmail.com (O.L.F.); 5 Programa de Pós-Graduação em Ciências da Saúde, Universidade de Brasília, Distrito Federal 70910-900, Brazil; 6 S-Inova Biotech, Porgrama de Pós-Graduação em Biotecnologia, Universidade Católica Dom Bosco, Campo Grande 79117-900, Brazil; 7 Departamento de Biofísica, Universidade Federal de São Paulo, São Paulo 04039-032, Brazil; lcf.lima@gmail.com (L.C.F.-L.); araujo.ronaldo@unifesp.br (R.C.A.); 8 Department of Life Science, Whitelands College, University of Roehampton, London SW15 4DJ, UK; yildiria@roehampton.ac.uk (A.Y.); richard.mackenzie@roehampton.ac.uk (R.M.)

**Keywords:** dietary management, exercise training, metabolism, adipokines

## Abstract

High-protein diets (HPDs) are widely accepted as a way to stimulate muscle protein synthesis when combined with resistance training (RT). However, the effects of HPDs on adipose tissue plasticity and local inflammation are yet to be determined. This study investigated the impact of HPDs on glucose control, adipocyte size, and epididymal adipose inflammatory biomarkers in resistance-trained rats. Eighteen Wistar rats were randomly assigned to four groups: normal-protein (NPD; 17% protein total dietary intake) and HPD (26.1% protein) without RT and NPD and HPD with RT. Trained groups received RT for 12 weeks with weights secured to their tails. Glucose and insulin tolerance tests, adipocyte size, and an array of cytokines were determined. While HPD without RT induced glucose intolerance, enlarged adipocytes, and increased TNF-α, MCP-1, and IL1-β levels in epididymal adipose tissue (*p* < 0.05), RT diminished these deleterious effects, with the HPD + RT group displaying improved blood glucose control without inflammatory cytokine increases in epididymal adipose tissue (*p* < 0.05). Furthermore, RT increased glutathione expression independent of diet (*p* < 0.05). RT may offer protection against adipocyte hypertrophy, pro-inflammatory states, and glucose intolerance during HPDs. The results highlight the potential protective effects of RT to mitigate the maladaptive effects of HPDs.

## 1. Introduction

High-protein diets (HPD) have been increasingly popular in society owing to their association with enhanced satiety and energy expenditure, and thus they are used as an effective strategy in weight loss interventions during calorie restriction [1,2]. Indeed, reduced body weight and improved health outcomes, such as reductions in fasting insulin, waist circumference, and total fat mass, are displayed in obese individuals following an HPD intervention [3]. In addition, resistance-trained (RT) individuals often use HPDs to promote lean mass and muscle protein synthesis [4]. However, high protein intake alone does not seem to facilitate muscle strength improvements, although it is associated with improved body composition [4,5].

A recent meta-analysis has shown that the effects of HPDs, such as reducing adiposity, glucose levels, and lipid profile, are insignificant [6] and suggests that long-term effects of HPDs, when not combined with RT, are still controversial. Although HPDs mitigate obesity effectively in animal studies [7,8], elsewhere some evidence suggests a possible harmful effect of high protein ingestion [9,10], with Lima et al. [9] reporting that HPDs resulted in impaired blood glucose control and lipid profile parameters and enlarged liver and kidney weight in sedentary rodents. More recently, Nogueira et al. [10] demonstrated that HPDs increased IL-6 levels in the left ventricle; moreover, RT was not sufficient to alleviate this pro-inflammatory response but did reduce cardiac TNF-alpha levels. Taken together, these data present a confusing picture of the possible health benefits associated with HPDs. Despite these advances, the effects of HPDs when combined with RT in different tissue types, such as in adipocytes, is yet to be fully determined.

Visceral adipose tissue has a critical function for remodeling responses to different conditions, including weight loss/gain [11], increased or decreased intake of nutrients [12], as well as exercise training [13,14]. Clinical trials and animal models demonstrated that adipose tissue dysfunction is defined by oxidative stress, low-grade inflammation, reduced insulin-mediated inhibition of lipolysis, and adipocyte hypertrophy [15,16,17]. The rise in high-calorie dietary intake and calorie-unbalanced well-known risk factors negatively affect adipose tissue function [18]. Still, the plasticity and molecular mechanisms in specific tissues remain unclear during HPDs.

Exercise training is a potent mediator of lipolysis, while also preventing rapid fat expansion and consequently attenuating inflammatory response in parallel with improved insulin sensitivity [19]. Some reports suggest that exercise is a promising non-pharmacological strategy for improving lipid and glucose metabolism, in addition to promoting the release of several adipokines and normalizing the redox imbalance in the adipose tissue [20,21]. RT has a prophylactic effect by suppressing pro-inflammatory factors (i.e., TNF-α, IL-6, IL1-β, and MCP-1) and enhancing antioxidant enzymes (i.e., reduced glutathione) and adiponectin, which may be crucial for adipose tissue homeostasis and metabolic health [22,23]. Presumably, studies investigating these molecules might provide valuable insight and clues for dietary management and might clarify relevant protective mechanisms involved in diets with different protein contents associated with exercise training. Thus, this study aimed to explore the impact of HPDs on glucose control, adipocyte size, and inflammatory cytokines in the visceral adipose tissue in rodents undertaking RT. 

## 2. Materials and Methods

### 2.1. Animals and Study Design

This study was carried out following international standards and recommendations and conformed with international principles for research involving animals (ARRIVE 2.0) and the National Animal Experiment Control Council (CONCEA, Brazil), in compliance with the Guide for the Care and Use of Laboratory Animals. The study protocol was approved by the Federal University of Mato Grosso do Sul animal ethics committee (protocol number 854/2017). Eighteen Wistar rats (~45 days, ~199g) were used to ensure the minimum number of animals viable for the investigated outcomes. Animals were housed in collective cages (*n* = 2 or 3) at 21 °C on a 12:12 light-dark cycle and had access to water. Animals were randomized into four groups: normoprotein (NPD, 17% of energy from protein) control without exercise (NPD-C, *n* = 5), NPD received RT (NPD-RT, *n* = 5), high-protein diet (HPD, 26.1% of energy from protein) control without exercise (HPD-C, *n* = 4) and HPD received RT (HPD-RT, *n* = 4). The diet was adapted from Reeves et al. [24], and the casein was replaced by whey protein (80%) [25], and all groups received an isocaloric diet. The diet and exercise intervention lasted 12 weeks. Food consumption was assessed weekly, and the energy metabolized from macronutrients was calculated without taking fiber into account. After the last procedure, the animals were euthanized by intraperitoneal injection (ketamine/xylazine). The visceral adipose tissue sites were used, which were removed and stored at −80 °C for further analysis. An experienced researcher dissected all depots to prevent cross-contamination with other tissues.

### 2.2. Resistance Training Protocol

Initially, animals were familiarized with the device (vertical ladder, 1.1 m × 0.18 m, 2 cm grid, 80° incline) without any load and were next subjected to an incremental test to determine the maximum load. The rats completed three sections of familiarization with 48 h intervals. After the familiarization process, the maximum load of individual animals was assessed. Animals completed 4–9 stairway climbs with loads gradually interspersed with a 120 s interval per effort [26,27]. The beginning climb was performed at 75% of body mass. Upon conclusion of this load, an extra 30 g weight was attached to the apparatus until the rats could not climb the complete steps.

The resistance training protocol was performed for 12 weeks, three times per week in the afternoon period, which was carried out with four progressive climbing series, beginning with 50%, 75%, 90%, and 100% of the highest load obtained in the incremental test [28]. After the 100% load, 30 g was added for the animal to perform another session until unable to perform the movement. The animals rested 2 min between each set. The load was readjusted weekly to guarantee the overload principle. The animals in the control group were kept in cages during the exercise training period.

### 2.3. Metabolic Analysis

After 24 h of the last RT session, animals were fasted for 6 h for the glucose tolerance test ipGTT (2 g/kg; glucose 50%) and were monitored for 2 hours after the glucose infusion. On the next day, the intraperitoneal insulin tolerance (ipITT) test was performed with standard human insulin (Biohulin, Brazil) administrated at 1.5 IU/kg, and glucose was monitored for 1 h. Glucose concentrations were measured in duplicate (Accu-Check Advantage. Roche^®^ Indianapolis, Indianapolis, IN, USA) to determined area under the curve (AUC, (mg/dL × 120 min)) and glucose decay index (kITT, %/min) by linear regression, which represents the decline in glucose levels, according Bonora et al. [29]. All analyses were performed using whole blood drawn from the tail.

### 2.4. Histological Analysis

Adipose tissue (epididymal site) was fixed in 10% neutral-buffered formalin (VWR, Mississauga, ON, Canada) dehydrated in xylene (Fisher Scientific, Waltham, MA, USA) and embedded in paraffin at 60 °C. The sections of 8 mm cut on the microtome (Leica, Wetzlar, Germany) were stained by hematoxylin and eosin (HE, Sigma Aldrich, St. Louis, MO, USA) for determining adipocyte cross-sectional area (CSA). Approximately 100 cells from each animal were used to determine the cross-sectional area (ImageJ software; National Institute of Mental Health, Bethesda, MD, USA) according to Macpherson et al. [30]. Pictures were acquired using an Olympus BV51 microscope equipped with an SV Micro Sound Vision digital camera (Preston South, Australia) at 100× magnification. The CSA analysis experiments were conducted by a blinded researcher, attenuating possible bias related to this process.

### 2.5. Biomarkers Analysis

Glutathione (GSH) levels were measured in the epididymal site of adipose tissue samples following homogenation. After mixture and incubation with 12.5% trichloroacetic acid (TCA), the GSH was measured spectrophotometrically using DTNB (5, 50 dithiobis-2-nitro-benzoic acid, Sigma, St. Louis, MO, USA), as previously described [31].

A multiple analysis kit was obtained from Linco Research Inc. (St. Charles, MO, USA). Millipore multiscreen 96-well filter plates were used with all multiplex kits. Adiponectin, IL-6, MCP-1, IL-1β, and TNF-α levels were determined by enzyme-linked immunosorbent assay (ELISA), using the respective rat ELISA development kit (RADPCMAG-82K-05). Adipokine levels were shown as picogram per milliliter (pg.mL) after agreement with the standard curve suggested by the ELISA kit, which was applied following the manufacturer’s stipulations. All samples were prepared in duplicate to ensure reliability. The minimum detectable level was 0.33 pg.mL. The intra-assay coefficient of variation was 0.37–1.02%, and the inter-assay coefficient of variation was 0.06–3.82%. Data were obtained utilizing the Luminex-200 system Version 1.7 (Luminex, Austin, TX, USA).

### 2.6. Statistical Analysis

Results are expressed as means ± standard deviation (SD). Shapiro–Wilk and Levene’s tests were used to analyze the data normality and the homogeneity of the variance. A one-way or two-way independent ANOVA (Exercise × Diet or Time × Groups) was used to compare adipocyte size, biomarkers in epididymal site of adipose tissue, and glucose control. A Bonferroni post hoc test was applied to identify the differences when a significant difference was found. A α level of 5% and power (1 − β = 0.9) was considered for this experiment. GraphPad Prism 7.0 (San Diego, CA, USA) and G*Power 3.1 were used for statistical analysis and graphics design.

## 3. Results

### 3.1. Diet Characteristics

Table 1 shows the result from diets. There were significant differences in food intake (*p* = 0.0003) and consequently in calorie intake (*p* = 0.0002), but without affecting the food efficiency between the groups (*p* > 0.05) at the end of 12 weeks.

### 3.2. Body Weight Gain and Strength Development

**Figure 1:**
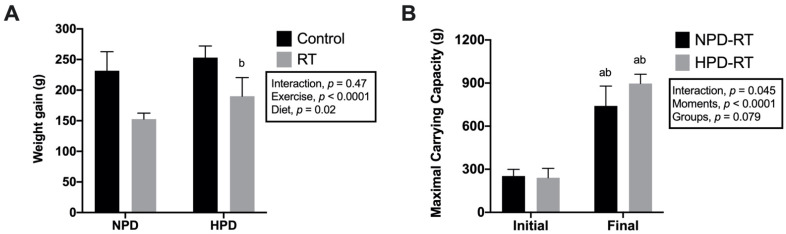
Body weight gain and strength development. (**A**) Body weight gain; (**B**) maximal carrying capacity. Values are presented as means ± SD. ^a^ *p* < 0.05 vs. NPD-Control, ^b^ *p* < 0.05 vs. HPD-Control.

Importantly, weight gain was significantly lower in resistance-trained groups compared with their controls (*p* < 0.0001, Figure 1A). Although the animals increased strength over the 12 weeks of RT (*p* < 0.05), no differences were observed between the maximum strength between the trained groups (NPD-RT vs. HPD-RT) after 12 weeks of RT (*F*_exercise_ (1, 16) = 3.501, *p* = 0.08) (Figure 1B).

### 3.3. Whole-Body Glucose and Insulin Tolerance Tests

**Figure 2:**
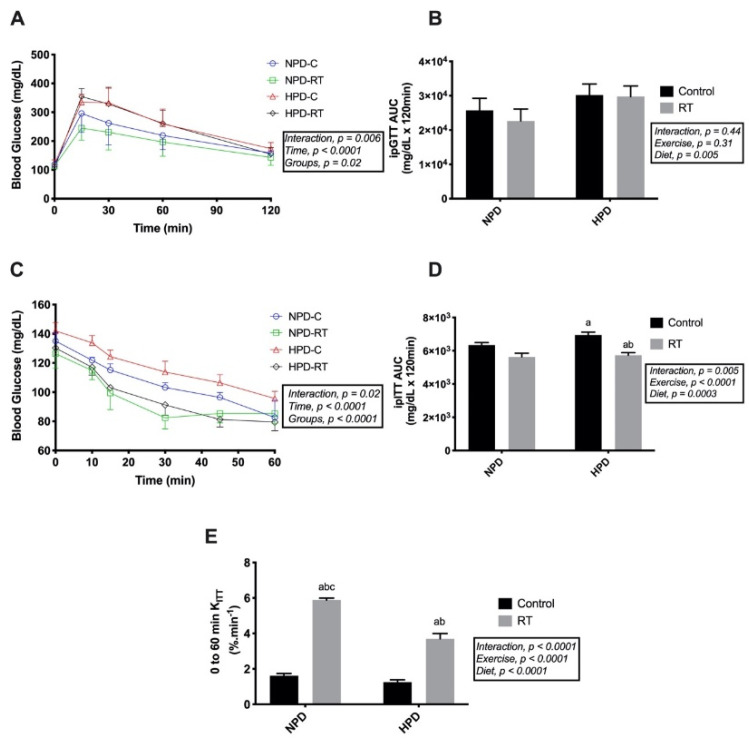
Whole-body glucose and insulin tolerance tests. (**A**) Blood glucose values throughout an insulin tolerance test; (**B**) the total area under curve ipGTT; (**C**) blood glucose values throughout a glucose tolerance test; (**D**) the total area under the curve ipITT; (**E**) glucose decay index. Values are presented as means ± SD. ^a^ *p* < 0.05 vs. NPD-Control, ^b^ *p* < 0.05 vs. HPD-Control.

Two-way ANOVA demonstrated an interaction between groups and time for ipGTT (*F*_interaction_ (12, 64) = 2.670, *p* = 0.006). Regarding the groups and ipGTT, the NPD-RT group showed differences in relation to HPD-C (*p* = 0.02) and HPD-RT (*p* = 0.008) at the 15th minute (Figure 2A). After the ipGTT, glucose kinetics revealed that only the NPD-RT group showed a reduction in AUC compared with high-protein groups (NPD-RT vs. HPD-C *p* = 0.03 and vs. HPD-RT, *p* = 0.04) (Figure 2B). Concerning the insulin tolerance test, a significant interaction was identified (*F*_interaction_ (15, 96) = 2.104, *p* = 0.0158). Accordingly, the HPD-RT group showed differences between all-time points with its HPD-C control (Figure 2C, *p* < 0.05). RT decreased AUC_glu_ for NPD-RT and HPD-RT when compared with the control groups during the iTT (Figure 2D, *F*_interaction_ (1, 16) = 10.56, *p* = 0.005). Further, the _K_ITT (0 to 60 min) showed a significant increase in glucose clearance in the trained groups (NPD-RT, 5.88 ± 0.11%/min, HPD-RT, 3.70 ± 0.3%/min) compared with the controls (NPD-C, 1.61 ± 0.13%/min, HPD-C, 1.25 ± 0.12%/min). However, HPD-RT (3.70 ± 0.3%/min) differed from NPD-RT (5.88 ± 0.11%/min) (*p* < 0.0001) (Figure 2E, *F*_interaction_ (1, 12) = 98.22, *p* < 0.0001.

### 3.4. Effects of HPD Diet and RT on Adipose Tissue Weight and Adipocyte Size

**Figure 3:**
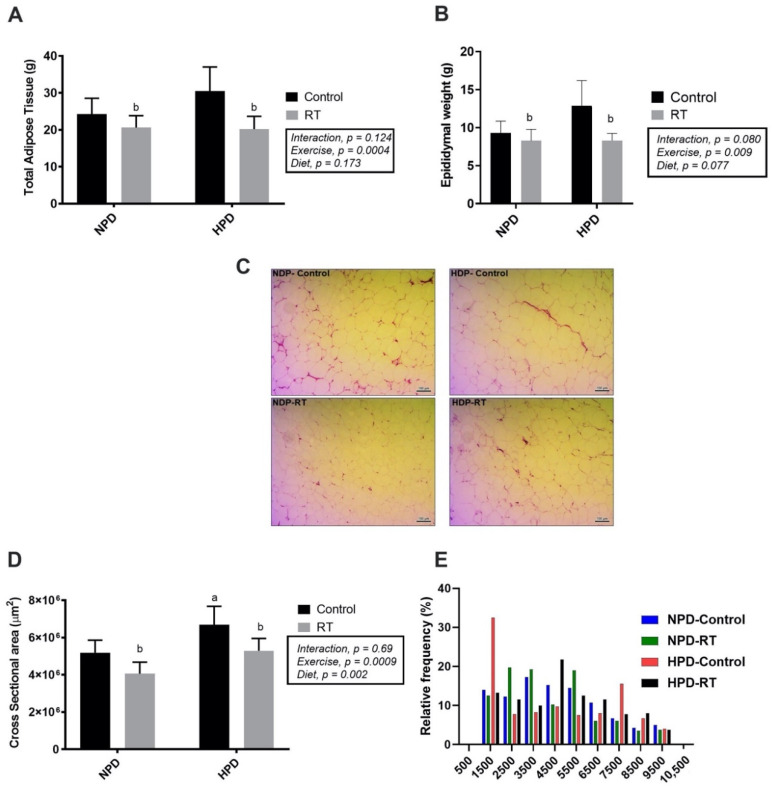
Adipose tissue weight and adipocyte size. (**A**) Total visceral adipose tissue weight; (**B**) epidydimal adipose tissue weight; (**C**) histological sections of the epidydimal adipose tissue post-operation hematoxylin-eosin staining; (**D**) cross-sectional area of epidydimal adipocytes; (**E**) histogram of frequency distribution adipocyte size. Values are presented as means ± SD. ^a^ *p* < 0.05 vs. NPD-Control, ^b^ *p* < 0.05 vs. HPD-Control.

The resistance-trained groups displayed a reduction in total visceral adipose tissue and epididymal weight compared with the HPD group (*p* = 0.03 and *p* = 0.0007; Figure 3A, B). Histological analysis of epididymal adipose tissue (Figure 3C) showed that adipocyte cross sectional area (CSA) was significantly increased in HPD-C (6.7 × 10^6^ ± 9.9 × 10^5^ μm^2^) when compared with the NPD-C group (5.2 × 10^6^ ± 6.7 × 10^5^ μm^2^) (*F*_diet_ (1, 16) = 16.51, *p* = 0.0009). However, both NPD-RT (4.1 × 10^6^ ± 6.2 × 10^5^ μm^2^) and HPD-RT (5.3 × 10^6^ μm^2^) presented with reduced adipocyte CSA in comparison with the non-exercising high-protein diet group (HPD-C) (*F*_exercise_ (1, 16) = 14.04, *p* = 0.0018) (Figure 3D). The NPD-RT group had decreased frequency of large adipocytes (distribution ranging from 6500 to >9500 μm^2^) when compared with all groups. Adipocytes measuring 1500 µm^2^ or less represented 32% of all adipocytes in HPD-C, whereas they represented only 13% of all adipocytes in HPD-RT group. Moreover, the frequency of 7500 μm^2^ adipocytes was higher in HPD-C relative to the HPD-RT group. This indicates that RT decreased the frequency of larger adipocytes in the HPD condition (Figure 3E).

### 3.5. Cytokines and GSH Levels in the Visceral Adipose Tissue in Response to HPD Diet and RT

**Figure 4:**
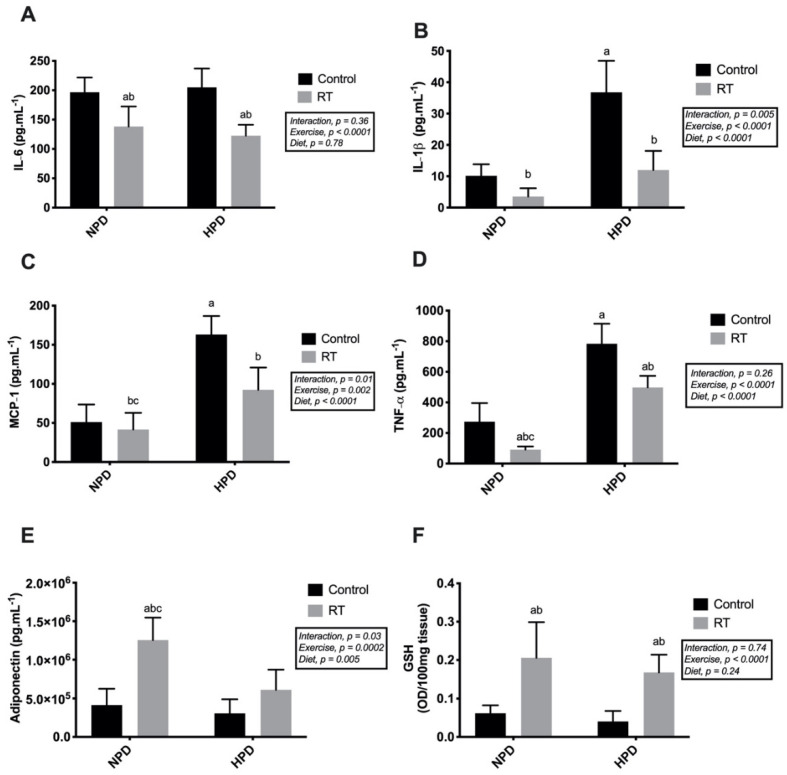
Inflammatory responses of epidydimal adipose tissue after 12 weeks of the experiment. (**A**) IL-6; (**B**) IL1-β; (**C**) MCP-1; (**D**) TNF-α; (**E**) adiponectin; (**F**) GSH. Values are presented as means ± SD. ^a^ *p* < 0.05 vs. NPD-Control, ^b^ *p* < 0.05 vs. HPD-Control, and ^c^ *p* < 0.05 vs. HPD-RT.

HPD-C displayed significantly higher TNF-α concentration compared with the NPD-C group (*F*_diet_ (1, 16) = 110.0, *p* < 0.0001). However, RT reduced TNF-α concentration for both NPD-RT and HPD-RT when compared with controls (Figure 4A, *p* < 0.0001). RT also reduced IL-6 (Figure 4B) in adipose tissue when compared with normal feeding and high-protein feeding controls (*F*_exercise_ (1, 16) = 31.20, *p* < 0.0001). MCP-1 levels were higher under HPD conditions (Figure 4C); although RT reduced MCP-1 levels, expression of MCP-1 (41.5 ± 21.3 pg.mL^−1^) was lower in NPD-RT when compared with HPD-RT (92.4 ± 28.5 pg.mL^−1^) (*F*_interaction_ (1, 16) = 8.024, *p* = 0.012). Furthermore, expression of IL-1β was higher in HPD-C when compared with the other groups. Consequently, RT reduced the levels of IL-1β (NPD-RT, 3.53 ± 2.67 pg.mL^−1^; HPD-RT, 11.99 ± 6.11 pg.mL^−1^) in the HPD-C group (36.81 ± 10.05 pg.mL^−1^) (*F*_interaction_ (1, 16) = 10.44, *p* = 0.005) (Figure 4D).

Regarding adiponectin levels in adipose tissue, only the NPD-RT (1.25 × 10^6^ ± 2.93 × 10^5^ pg·mL^−1^) showed a significant increase after 12 weeks of intervention compared with NPD-C (4.14 × 10^5^ ± 2.12 × 10^5^ pg.mL^−1^), HPD-C (3.07 × 10^5^ ± 1.83 × 10^5^ pg.mL^−1^), and HPD-RT (6.13 ± 2.62 pg.mL^−1^) (*F*_interaction_ (1, 14) = 5.728, *p* = 0.031) (Figure 4E). Finally, 12 weeks of RT significantly increased GSH compared with control groups (NPD-RT, 0.21 ± 0.09 OD/100 mg tissue; HPD-RT, 0.17 ± 0.05 OD/100 mg tissue vs. NPD-C, 0.06 ± 0.02 OD/100 mg tissue; HPD-C, 0.04 ± 0.03 OD/100 mg tissue) (*F* (1, 16) = 31.15, *p* < 0.0001) (Figure 4F).

## 4. Discussion

**Figure 5:**
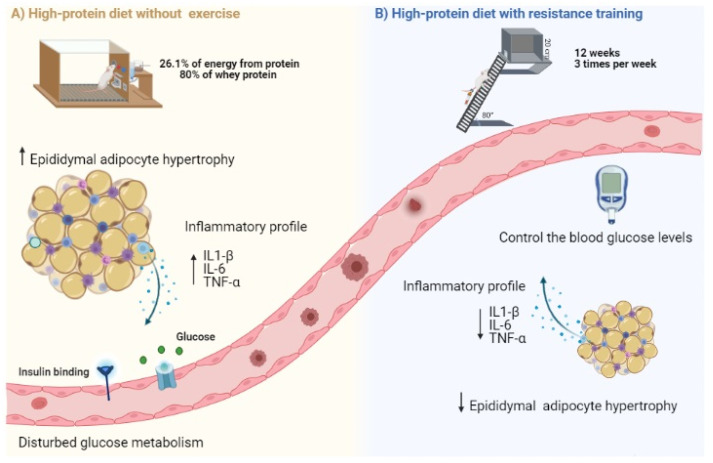
Overview of the effects of high-protein diet and resistance training on glucose control, adipocyte size, and epididymal adipose inflammatory biomarkers in rats. (**A**) High-protein diet without exercise induced epididymal adipocyte hypertrophy and increased TNF-α, MCP-1, and IL1-β levels, which combined may have contributed to disturbed glucose metabolism; (**B**) Resistance exercise training compensated the detrimental effects of high-protein diet by reducing epididymal adipocyte hypertrophy, local inflammation, and glucose blood levels. The figure was created in the Biorender web-based software.

This study demonstrates that HPD without resistance exercise increases adipocytes’ CSA and local proinflammatory cytokines. It is hypothesized that these two findings combined may have contributed to the impaired whole body glucose control seen in our study (Figure 5). Interestingly, RT appeared to attenuate the protein-induced adipocyte hypertrophy and proinflammatory state in adipose tissue. These findings were accompanied by improved glucose in the HPD-RT group compared with HPD-C. Our results provide support to the potential negative impact of HPDs on protein-induced adipose inflammation and glycemic control in healthy rats. Combined, these data suggest that this training modality could mitigate potential issues caused by high protein ingestion.

In this study, whey protein was the main protein source in HPDs, which contains a high concentration of branched-chain amino acids (BCAA). Newgard [32] reported that lipids and BCAAs support the synergy of impaired insulin sensitivity and glucose utilization. Consequently, HPD exhibits insulinotropic traits, which in the long term can develop insulin resistance [33]. Although skeletal muscle plays a fundamental role in glucose homeostasis, other organs also play a crucial role. In this sense, adipose tissue has the endocrine function of controlling hormones associated with metabolism, such as glucose and lipid homeostasis. Therefore, dysregulation in adipose tissue can provoke a vital disturbance in glycemic control [34], which may partially explain the findings in the AUC of the ipTTG and ipITT of the HPD-C group in the present study. Accordingly, our findings indicate that HPDs are associated with undesirable effects in glucose control in a healthy rodent model and that the negative effects described in our data are seemingly reversed with RT.

The HPD-RT group demonstrated a reduction in AUC_glu_ in response to the insulin tolerance test, which can be attributed to the well-documented increase in glucose uptake in skeletal muscle in response to exercise training [35]. Protein ingestion and amino acid availability are known to stimulate muscle protein synthesis [36], while protein consumption also generates a higher thermic cost compared to lipids and carbohydrates, particularly when combined with resistance exercise training [37]. Morato et al. [38] demonstrated that whey protein promotes higher GLUT-4 and Akt expression in exercised rodent muscle cells when compared with casein protein ingestion. In addition, RT and high-intensity exercise are also known to enhance the expression of key regulatory proteins AktThr^308^ and AS160Thr^642^ in the skeletal muscle [35,39]. Consequently, resistance exercise promotes improved glucose control by AMPK activation and the mTOR signaling pathway, increasing the concentration of GLUT-4 translocation [40,41].

Adiponectin is a peptide secreted via adipocytes, which have anti-inflammatory and anti-atherogenic effects [42,43]. Increased adiponectin levels have been linked with reduced risk of cardiovascular disease, type 2 diabetes, and obesity [42,43]. Currently, adiponectin levels serve as a critical biomarker targeting metabolic diseases [43]. Our results indicate an increase in adiponectin levels in the NPD-RT group compared with the other groups. Nevertheless, the RT intervention was not able to increase adiponectin in rats receiving HPD. Although adiponectin was only measured in adipose tissue, these results may indicate a potentially harmful effect of HPD. There is some evidence that excess protein could be converted to glucose and stored as glycogen and fat [1,2], which could explain adipocyte hypertrophy in the HPD group, as well as lower adiponectin levels in HPD-RT when compared with NDP-RT. Moreover, adipocyte hypertrophy induces cellular dysfunction and promotes pathological conditions, including insulin resistance [44].

Studies examining the frequency distribution of adipose cells showed that adipocytes are not homogeneous in their ability to uptake lipids or their functional properties in response to a high-fat diet [45]. Ibáñez et al. [46] explained that asymmetry in cell distribution is a critical outcome of proliferation and differentiation since small adipocytes are linked to an increased rate of adipocyte proliferation, and large adipocytes are associated with increased lipid storage capacity. The novel findings from this study are that HPD-Control displayed a nearly bimodal distribution of adipose cells, which suggests that high-protein diets associated with sedentarism can increase both mature and immature cells or cells incapable of maximally depositing triglyceride.

Furthermore, HPD upregulates classic inflammatory cytokines, such as TNF-α, MCP-1, and IL-1β in visceral adipose tissue. These findings are compatible with previous studies, which suggest that these inflammatory signals contribute to adipocyte dysfunction and impairment of insulin signaling [47,48]. On the other hand, RT was able to alleviate the inflammatory response in HPD groups, which implies that RT could be a protective factor against the undesired effects of HPDs. Lira et al. [49] reported that exercise training significantly downregulated pathways involved in this inflammatory cycle (TLR-4 and NF-kβp65), and that exercise-induced activation of peroxisome proliferator-activated receptor alpha (PPAR-α) ameliorates inflammatory response in adipose tissue. Moreover, we observed that RT also reduced IL-6 levels in both resistance-trained groups, which is a potent cytokine responsible for macrophage accumulation in visceral adipose tissue and might be an important additional contributor to the inflammatory response [50].

It is reported that ROS have been playing a significant function in FFA-induced insulin resistance by insulin receptor substrate-1 phosphorylation in serine residue and by inhibiting downstream insulin signaling [51]. Consequently, adipocytes operate in new, higher redox equilibrium, restricting the release of key antioxidant enzymes under nutritional overload [51,52], which might explain similar GSH levels in the HPD-C compared with the NPD-C group. On the other hand, we found that RT was able to increase GSH expression. The elevation observed in the resistance-trained groups might indicate the decomposition of reactive oxygen species and a presumable normalization of the tissue adipose redox state, which may have contributed to glucose metabolism improvements in the HPD-RT group. Overall, the current results provide potential evidence that RT is capable of orchestrating protective factors that might help to restore oxidative damage and glucose disturbance.

Several limitations of our study should be noted, including the inability to analyze insulin levels in the basal state, anti-inflammatory cytokines, and other critical proteins involved in molecular processes mediating oxidative metabolism. Additionally, only the epididymal site was evaluated in this study, which leaves us unable to draw conclusions about specific adaptations or other adipose tissue sites. There is also evidence that suggests heterogeneous adipokine levels in different adipose depots after exercise [53]. Future studies should investigate the ability of RT to modulate other tissues, and we state the limitation here and look forward to the results of further studies. Finally, our results are also limited to the length of time of our diet exposure (i.e., 12 weeks) and to animal models; future human studies may be warranted to investigate this potential association between glucose metabolism and adipocyte inflammation.

## 5. Conclusions

In summary, in health rats, HPD resulted in impaired glucose tolerance, adipocyte hypertrophy, and increased inflammatory state in visceral adipose tissue at the end of 12 weeks. However, RT compensated for the detrimental effects of HPDs by normalizing blood glucose levels and reducing adipose tissue inflammation in HPD. These results suggest that resistance training has a protective effect against potentially harmful effects of HPDs. Furthermore, RT upregulates reduced glutathione activity in the adipose tissue regardless of diet, which has crucial importance in normalizing the redox imbalance. Therefore, the present study presents relevant insights into the molecular mechanisms implicated in nutrition combined with resistance training in a non-pathological model.

## Figures and Tables

**Table 1:** Characteristics of diets concerning experimental groups.

Diet Outcomes	NPD-C	HPD-C	NPD-RT	HPD-RT
Daily food intake (g)	20.1 ±0.4	20.7 ±0.1	19.5 ±0.5 ^b^	18.6 ±1.0 ^a,b^
Dietary metabolizable energy (kcal/day)	65.5 ±1.4	68.6 ±0.4 ^a^	63.3 ±1.3 ^b^	61.2 ±3.2 ^a,b^
Feed efficiency (%)	16.5	16.3	14.6	14.5

Dietary characteristics. Values are presented as means ± SD. ^a^ *p* < 0.05 vs. NPD-Control, ^b^ *p* < 0.05 vs. HPD-Control.
